# Comparative Investigation of 0.5Li_2_MnO_3_·0.5LiNi_0.5_Co_0.2_Mn_0.3_O_2_ Cathode Materials Synthesized by Using Different Lithium Sources

**DOI:** 10.3389/fchem.2018.00159

**Published:** 2018-05-15

**Authors:** Peng-Bo Wang, Ming-Zeng Luo, Jun-Chao Zheng, Zhen-Jiang He, Hui Tong, Wan-jing Yu

**Affiliations:** ^1^School of Metallurgy and Environment, Central South University, Changsha, China; ^2^College of Chemistry and Chemical Engineering of Xiamen University, Xiamen, China

**Keywords:** lithium-ion battery, lithium-rich manganese-based cathode materials, lithium sources, solid reaction, electrochemical performances

## Abstract

Lithium-rich manganese-based cathode materials has been attracted enormous interests as one of the most promising candidates of cathode materials for next-generation lithium ion batteries because of its high theoretic capacity and low cost. In this study, 0.5Li_2_MnO_3_·0.5LiNi_0.5_Co_0.2_Mn_0.3_O_2_ materials are synthesized through a solid-state reaction by using different lithium sources, and the synthesis process and the reaction mechanism are investigated in detail. The morphology, structure, and electrochemical performances of the material synthesized by using LiOH·H_2_O, Li_2_CO_3_, and CH_3_COOLi·2H_2_O have been analyzed by using Thermo gravimetric analysis (TGA), X-ray diffraction (XRD), Scanning electron microscope (SEM), Transmission electron microscope (TEM), X-ray photoelectron spectroscopy (XPS), and electrochemical measurements. The 0.5Li_2_MnO_3_·0.5LiNi_0.5_Co_0.2_Mn_0.3_O_2_ material prepared by using LiOH·H_2_O displays uniform morphology with nano particle and stable layer structure so that it suppresses the first cycle irreversible reaction and structure transfer, and it delivers the best electrochemical performance. The results indicate that LiOH·H_2_O is the best choice for the synthesis of the 0.5Li_2_MnO_3_·0.5LiNi_0.5_Co_0.2_Mn_0.3_O_2_ material.

## Introduction

Rechargeable Li-ion batteries (LIBs) play a dominant role in energy storage devices of portable electronic devices and electric vehicles (EVs) for its excellent safety, long cycle life, and high energy density (Armand and Tarascon, 2008; Goodenough and Park, 2013; Zhang Q. et al., 2015, 2016; Li et al., 2017a,b; Zhang et al., 2018). However, the specific energy density of LIBs still cannot meet the demand of EVs owing to lower energy density of cathode material (Whittingham, 2014). At present, classic cathode materials, such as LiFePO_4_ (Zheng et al., 2008, 2017), LiMn_2_O_4_ (Kim et al., 2008), LiCoO_2_ (Kang et al., 1999), and LiNi_a_Mn_b_Co_1−a−b_O_2_ (Li et al., 2017) etc., offer a reversible discharge capacity less than 200mAh g^−1^. Recently, lithium-rich manganese-based cathode materials has been attracted enormous interests as a promising cathode material for next-generation LIBs because of high discharge capacity (more than 250mAh g^−1^) and low cost (Ohzuku et al., 2011).

Lithium-rich manganese-based cathode materials contain double component: one phase of Li_2_MnO_3_ with C2/m space group and the other phase of LiMO_2_ with R-3m space group. Because Mn^4+^ in Li_2_MnO_3_ phase cannot be oxidized any more, it possesses electrochemically inert. However, Li^+^ and O_2_ can be extracted from the TM (transition metal) layer and the lattice, respectively, which indicates that the Li_2_MnO_3_ phase is activated by initial charging process and forms an irreversible loss of Li_2_O (Yabuuchi et al., 2011). It is demonstrated that the high capacity of the materials originates from the oxygen escape from Li_2_MnO_3_ phase at high voltage (Yabuuchi et al., 2011). In addition, there are so many fatal disadvantages in lithium-rich manganese-based cathode materials, such as severe voltage fading during cycling (Zheng J. et al., 2015; Zhang T. et al., 2016) poor rate performance (Fan et al., 2015; Rozier and Tarascon, 2015; Zhang K. et al., 2015), large initial irreversible capacity, and low initial coulombic efficiency (Bai et al., 2015). Presently, many methods like comprising doping (Dianat et al., 2013; Wang et al., 2013; Li et al., 2014; Zhang H. et al., 2014), coating (Shi et al., 2012, 2013; Gu et al., 2013; Zhang et al., 2013; Zhou et al., 2017; Liu et al., 2018), nano crystallization (Wang et al., 2010), and morphology control (Yang et al., 2013; Remith and Kalaiselvi, 2014) have been proposed to promote the electrochemical performance of the materials.

Generally speaking, the compositions of this kind of material are varied. According to the chemical formula of lithium-rich manganese-based cathode materials, it can be simply written as *x*Li_2_MnO_3_·(*1-x*)LiMO_2_(M = Ni, Co, Mn, Ti, Fe, etc.; Ohzuku et al., 2011; Yabuuchi et al., 2011; Bai et al., 2015; Fan et al., 2015; Rozier and Tarascon, 2015; Zheng J. et al., 2015; Zhang T. et al., 2016). Common and representative components of lithium-rich manganese-based materials are 0.5Li_2_MnO_3_·0.5LiNi_0.5_Mn_0.5_O_2_ and 0.5Li_2_MnO_3_·0.5LiNi_1/3_Co_1/3_Mn_1/3_O_2_ (Shi et al., 2012, 2013; Dianat et al., 2013; Gu et al., 2013; Wang et al., 2013; Yang et al., 2013; Zhang et al., 2013; Zhang H. et al., 2014; Li et al., 2014; Zhou et al., 2017).

To the best of our knowledge, the presence and content of nickel that improves the cathode capacity (Sun et al., 2005). Zheng Z. et al. (2015) discussed that the roles and the functions of nickel in electrochemical cycling of lithium-rich Mn-based cathode materials. Yang et al. (2017) reported that the Ni substitution at 2c sites not only enhances oxygen stability and delays oxygen loss from the lattice but also suppresses the cation mixing that induces the undesired phase transition. Gao et al. (2017) demonstrated that Li-rich material Li_1.2_(Ni_0.25_Co_0.25_Mn_0.5_)_0.8_O_2_ was prepared by a novel core-shell structure, in which Ni element acts as stabilizing ions to inhibit the Jahn-Teller effect of active Mn^3+^. Based on the above considerations, lithium-rich manganese-based material 0.5Li_2_MnO_3_·0.5LiNi_0.5_Co_0.2_Mn_0.3_O_2_ is designed for the first time.

In fact, the cathode materials with different morphology and electrochemical performance can be achieved by using different lithium sources (Zhang B. et al., 2014; Cao et al., 2017). In this work, we; try to synthesize 0.5Li_2_MnO_3_·0.5LiNi_0.5_Co_0.2_Mn_0.3_O_2_ materials with nano-size particles by using different lithium sources (LiOH·H_2_O, Li_2_CO_3_ and CH_3_COOLi·2H_2_O). The solid state reaction mechanism is investigated and the effects of lithium sources on the morphology, structure and electrochemical performance are clarified.

## Experimental

### Material preparation

The 0.5Li_2_MnO_3_·0.5LiNi_0.5_Co_0.2_Mn_0.3_O_2_ cathode material was prepared *via* solid-state reaction. Analytical grade chemicals NiC_4_H_6_O_4_·4H_2_O (AR, 99.9%), CoC_4_H_6_O_4_·4H_2_O (AR, 99.5%), MnC_4_H_6_O_4_·H_2_O (AR, 99%), and different lithium sources, LiOH·H_2_O, Li_2_CO_3_, and CH_3_COOLi·2H_2_O (excess 3.33% molar ratio, AR 95%, AR 98%, AR 99%, respectively), with a stoichiometric amount were mixed thoroughly and ball milled (200 rpm) for 1 h. With an amount of ethanol added, the materials were ball milled (200 rpm) continually for 3 h and dried in an oven at 80°C for 12 h to obtain a uniform mixed precursor. Then the dried precursor was ball milled for 0.5 h and sintered at 900°C for 10 h in air atmosphere to prepare the targeted 0.5Li_2_MnO_3_·0.5LiNi_0.5_Co_0.2_Mn_0.3_O_2_ material. The rate of heating was retained at 5°C min^−1^. The 0.5Li_2_MnO_3_·0.5LiNi_0.5_Co_0.2_Mn_0.3_O_2_ compounds synthesized by using LiOH·H_2_O, Li_2_CO_3_, and CH_3_COOLi·2H_2_O as lithium sources are marked as Sample A, Sample B, and Sample C, respectively.

### Sample characterization

The crystalline structure of 0.5Li_2_MnO_3_·0.5LiNi_0.5_Co_0.2_Mn_0.3_O_2_ was tested by X-ray diffraction (XRD, Rigaku D/maxb) with Cu Kα radiation(λ = 1.54056Å) in the range of 10°-80° with the speed of 5° min^−1^. The morphology was investigated with scanning electron microscopy (SEM, Philips, FEI Quanta 200 FEG) and transmission electron microscopy (TEM, TECNAI G2 F20, FEI). The sample was examined by Thermo gravimetric/Differential Scanning calorimeter (TG/DSC, SDT Q600) under the air from ambient temperature to 1,000°C at 10°C min^−1^. X-ray photoelectron spectroscopy (XPS, VG Multilab 2000) was used to observe the chemical valence of the TMs (transition elements Ni, Co, Mn, O) of the sample.

### Electrochemical measurements

Electrochemical measurements of 0.5Li_2_MnO_3_·0.5LiNi_0.5_Co_0.2_Mn_0.3_O_2_ were tested by CR2025 coin-type cells. The positive electrode was operated as slurry by 80% active material (0.5Li_2_MnO_3_·0.5LiNi_0.5_Co_0.2_Mn_0.3_O_2_), 10% acetylene black, 10% polyvinylidene fluoride (PVDF), and N-methylpyrrolidone (NMP) solvent. Then the electrode slurry was cast on aluminum foil and dried at 120°C for 12 h under vacuum atmosphere. Typical active material areal loadings were about 1.2 mg cm^−2^. The cells were assembled in a filled argon glove box. Lithium metal was used as the anode and the separator was a Celgard 2500. The electrolyte utilized was a 1 M LiPF_6_ solution by mixtures of ethylene carbonate (EC) and dimethyl carbonate (DMC) with a volume ratio of 1:1. The Galvanostatic charge-discharge measurements were carried out using NEWARE CT-3008 battery testing system (Shenzhen, China) within the voltage range of 2.0–4.8 V at room temperature. Cyclic voltammetry (CV) measurements were conducted using CHI660D Electrochemical Workstation (Shanghai Chen Hua) at 0.1 mV s^−1^ between 2.0 and 4.8 V. Electrochemical impedance spectroscopy (EIS) of the cell was carried out using CHI660D Electrochemical Workstation (Shanghai Chen Hua) in the frequency range of 0.1 Hz−10 kHz, and the AC voltage was applied as 5 mV.

## Results and discussions

**Figure 1:**
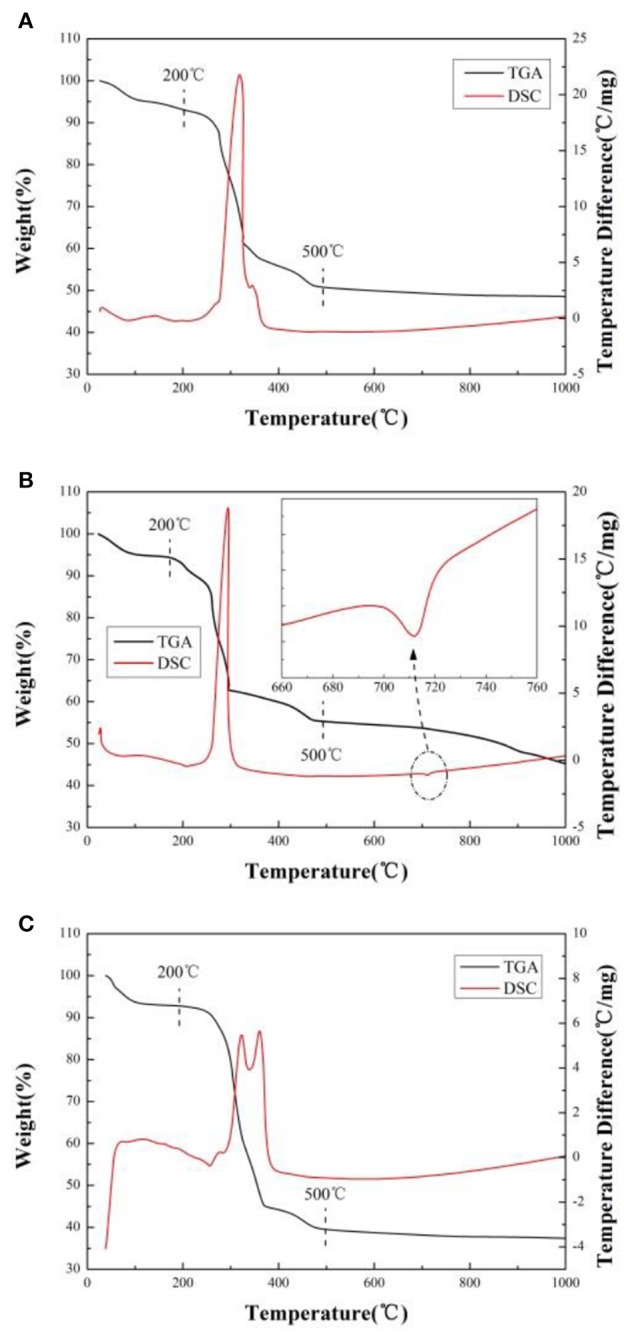
TG-DSC curves of the precursor by using different lithium sources. **(A)** LiOH·H_2_O, **(B)** Li_2_CO_3_, **(C)** CH_3_COOLi·2H_2_O.

In order to ascertain the optimum temperature for heat treatment and explore the effects of different lithium sources, TG-DSC analyses are done for the precursor in the air. As presented in Figure 1, there are three main stages for weight losses in the TG plots and several endothermic and exothermic peaks in the DSC plot. The temperature range from ambient to about 200°C, the weight loss is the release of hydration water from precursor (Deng et al., 2010). In the region from 200 to 500°C, there is a sharp exothermic peak (Figures 1A,B) or two exothermic peaks (Figure 1C) accompanied by abrupt weight loss observed in DTG/DSC curves, it should be related to the volatilization of crystallized water from MC_4_H_6_O_4_·4H_2_O(M = Ni, Co, Mn) and the decomposition of precursor. The weight loss of precursor mainly comes from the escape of water and carbon dioxide during the reaction. As the temperature increases from 500 to 1,000°C, weight loss (Figures 1A,**C**) almost can't be observed in DTG curves. However, a little exothermic peak and a small amount weight loss is observed in DSC curves (Figure 1B) when the temperature reaches 720°C, which corresponding to the melting temperature of lithium carbonate. We think that it attributes to carbon dioxide emission from lithium carbonate (Li_2_CO_3_).

The mass loss of sample A, B, and C is 51.44, 54.93, and 62.63%, respectively. The weight loss is from the decomposition of precursor and the release of water and carbon dioxide under heating (Cao et al., 2017). The greater the weight losses, the greater the quantity of gas releases (Cao et al., 2017). During the material formation under heat treatment, a large number of gas releases will destroy the primary particle morphology and promote it growth, resulting in aggregation. Because the mass loss of sample A is the least, the average size of particles and uniformity of the sample A should be much better than others. It will be proved in subsequent SEM images (Cao et al., 2017).

The XRD patterns for sample A synthesized at different temperatures are shown in Figure S1. It can be seen that the crystallinity of the samples increases accompanied by temperature increment from 600 to 1,000°C. As the temperature increases to 800°C, there appears a couple of peaks during 20–23° attributed to the super lattice diffraction of the monoclinic Li_2_MnO_3_ phase (Gao et al., 2017). With the temperature increasing to 900°C, the diffraction peaks of sample A are well indexed to a hexagonal α-NaFeO_2_ structure (Seteni et al., 2017). Sample A displays the (006)/(012) and (018)/(110) peaks with a fine splitting, it indicates that it possesses highly ordered good crystallinity layered structure. When the temperature reaches 1,000°C, the peak at 36.5° corresponding to LiMn_2_O_4_ with spinel structure. It indicates that LiMn_2_O_4_ structure can be formed at higher temperature (Zhang B. et al., 2014). Based on the above analysis, the optimum sintering temperature is chosen as 900°C in this work.

**Figure 2:**
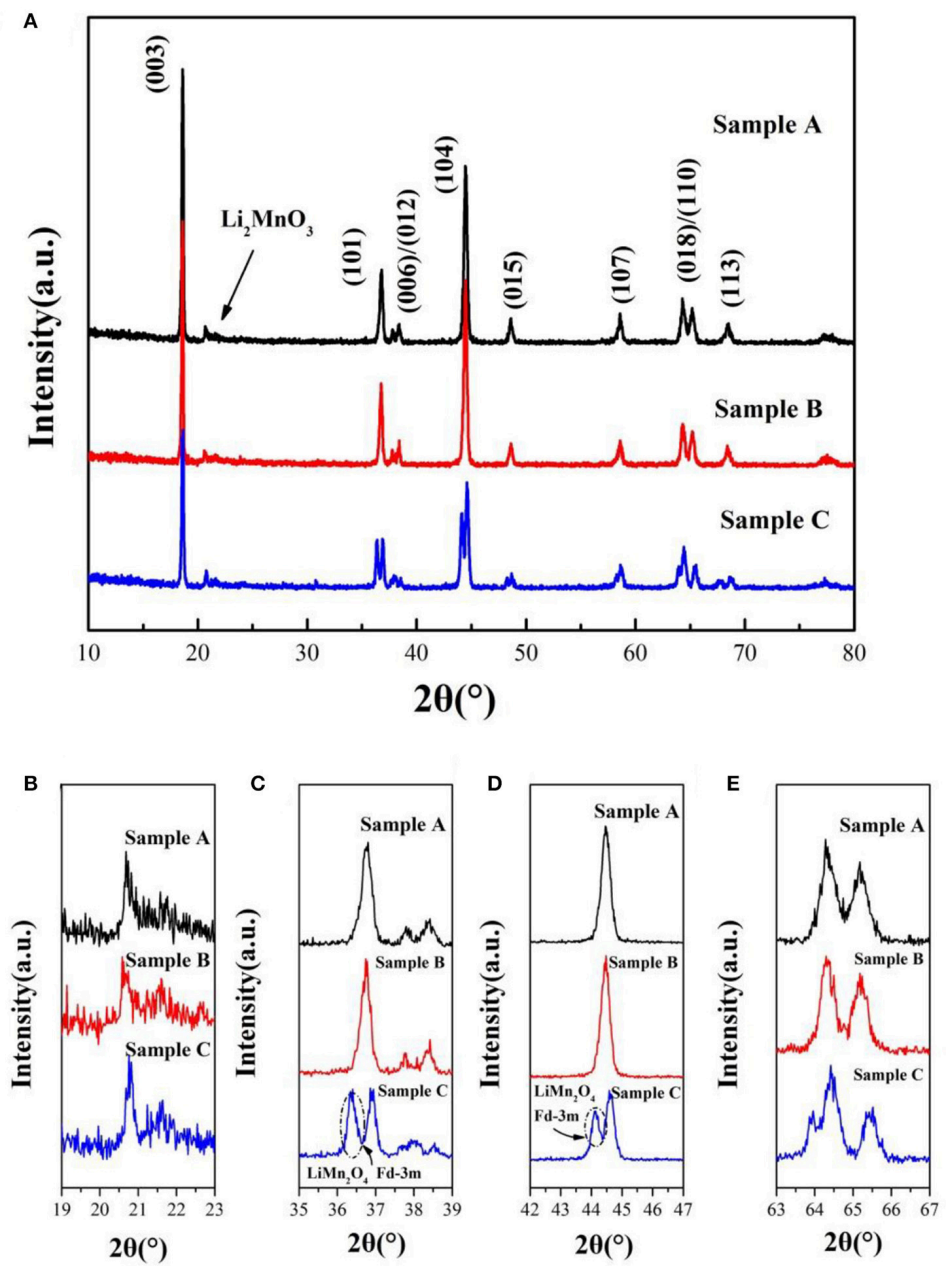
**(A)** XRD patterns of 0.5Li_2_MnO_3_·0.5LiNi_0.5_Co_0.2_Mn_0.3_O_2_ synthesized at 900°C by using different lithium sources and magnified XRD patterns in the 2θ range of **(B)** 19°–23°, **(C)** 35°–39°, **(D)** 42°–47°, **(E)** 63°–67°, respectively.

The XRD patterns of the sample A, B and C sintered at 900°C are shown in Figure 2. The peaks of the samples can be indexed to the hexagonal α-NaFeO_2_ phase (R-3m) and the little peaks from 20 to 23° (Figure 2B) are attributed to Li_2_MnO_3_ phase (C2/m). Li_2_MnO_3_ phase can be described as the ordering of lithium ions and transition metal ions in the layer for transition metal and the forming of LiMn_6_ arrangement (Gao et al., 2017). Besides, the two pairs of the (006)/(012) and (018)/(110) peaks are well separation, illustrating that the samples possesses good crystallinity and fine layered structure. Nevertheless, there are a group of minor peaks at 36.5° and 44° of sample C corresponding to LiMn_2_O_4_(Fd-3m) of formed under the high temperature. Lattice parameters of 0.5Li_2_MnO_3_·0.5LiNi_0.5_Mn_0.3_Co_0.2_O_2_ are listed in Table 1. The c/a ratios of samples are more than 4.9, it indicates that the material possesses layered characteristics (Zhang M. et al., 2016). The I_(003)_/I_(104)_ ratios of materials are much larger than 1.2, which indicates that the samples have low cationic mixing (Deng et al., 2010).

**Table 1:** Lattice parameters of 0.5Li_2_MnO_3_·0.5LiNi_0.5_Mn_0.3_Co_0.2_O_2_ synthesized by different lithium sources.

	**Lithium sources**	**a/Å**	**c/Å**	**c/a**	**I_003_/I_104_**
Sample A	LiOH·H_2_O	2.8643	14.2943	4.9905	1.5456
Sample B	Li_2_CO_3_	2.8610	14.2963	4.9970	1.3055
Sample C	CH_3_COOLi·2H_2_O	2.8671	14.3283	4.9975	1.4124

The SEM images of 0.5Li_2_MnO_3_·0.5LiNi_0.5_Co_0.2_Mn_0.3_O_2_ layered materials obtained before and after heat treatment are shown in Figure S2. The images of all samples possess morphology of similar aggregation, but sample A and B (Figures S2B,E) synthesized by LiOH·H_2_O and Li_2_CO_3_ have higher homogeneity. The sample C (Figures S2H,I) forms an irregular aggregation of primary particles. As shown in Figures S2B,E, sample A synthesized by LiOH·H_2_O shows much smaller primary particle sizes compared with other samples. The smaller particle size can shorten the diffusion distance of lithium ions and improve the electrochemical performance of the materials (Cao et al., 2017).

**Figure 3:**
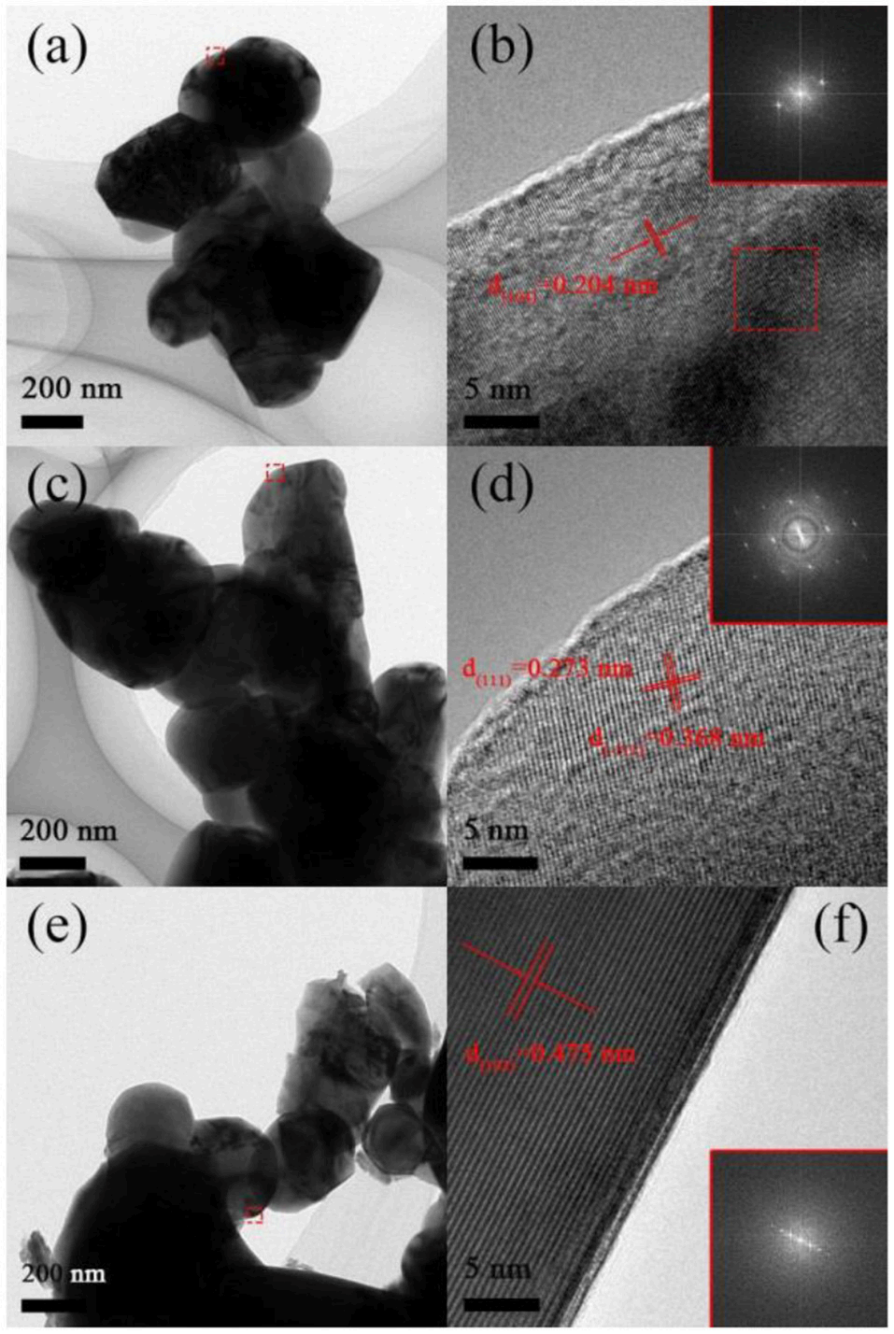
TEM images of **(A)** Sample A, **(C)** Sample B, **(E)** Sample C and HRTEM images of **(B)** Sample A, **(D)** Sample B, **(F)** Sample C.

To confirm SEM results, TEM analysis and the Fast Fourier Transform (FFT) are performed, as shown in Figure 3. Sample A composed of 200–300 nm primary particles and the distance of the lattice fringes of particles is calculated to be 0.204 nm, matching well with the d_(104)_ planes, which attributed to layer structure R-3m (Yang et al., 2016). Figures 3C,D show that sample B composed of 300–400 nm primary particles and the lattice spacing are 0.273 and 0.368 nm, corresponding to the planes d_(111)_ and d_(−111)_ of Li_2_MnO_3_ phase (C2/m) (Luo et al., 2014). As illustrated in Figures 3E,F, Sample C has severe aggregation although it possesses small primary particles from some regions owing to the destruction of released gas. The distance of the lattice fringes of this material is calculated to be 0.47 nm, corresponding to the d_(003)_ planes of layer structure (Yang et al., 2016).

**Figure 4:**
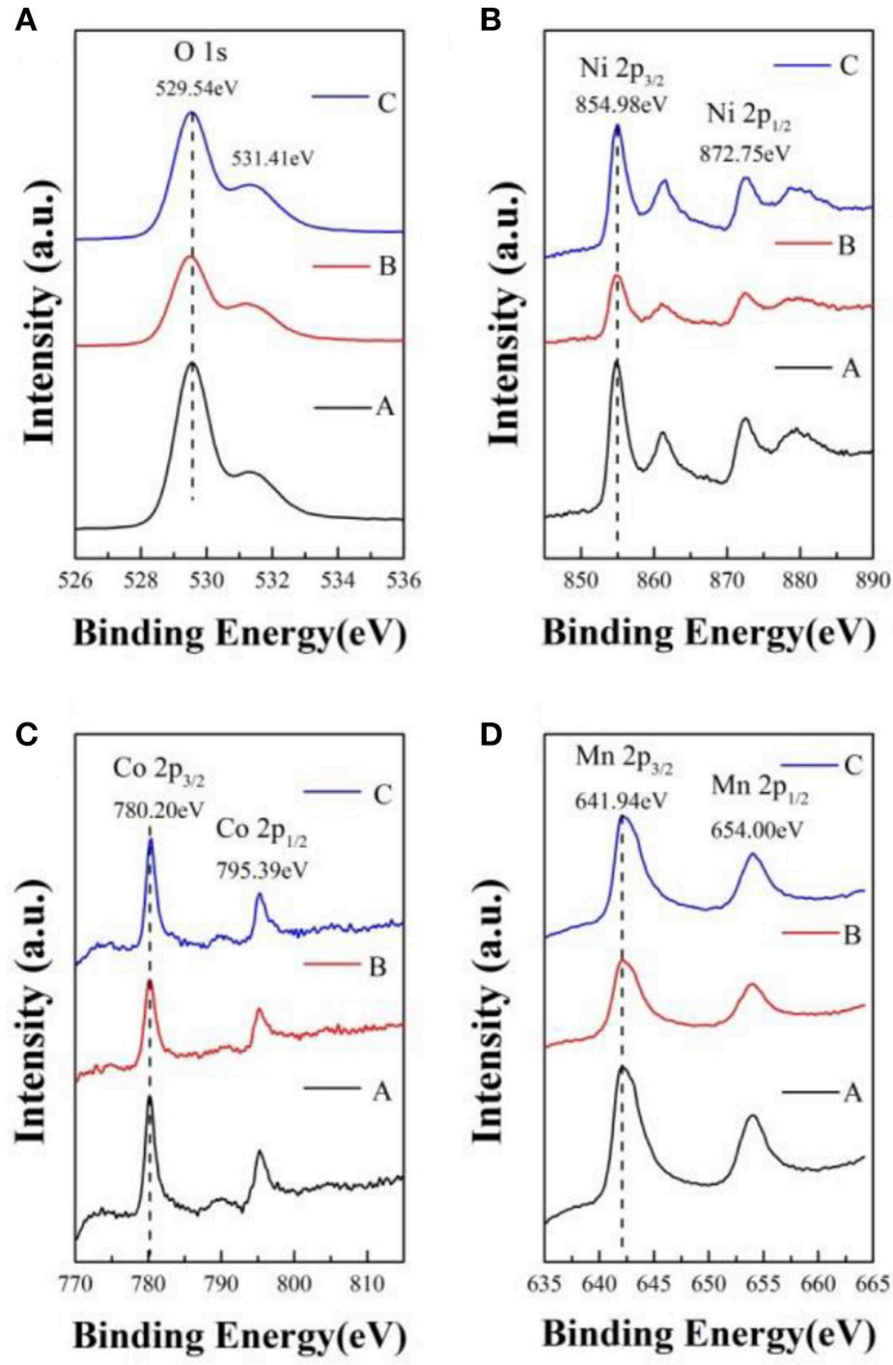
X-ray photoelectron spectra of sample A, B, and C [**(A)** O, **(B)** Ni, **(C)** Co, **(D)** Mn].

In order to determine the chemical valence of major elements, the X-ray photoelectron spectra of sample A, B, and C is analyzed and shown in Figure 4. In contrast with the samples, the O 1s, Ni 2p, Co 2p, and Mn 2p peaks have no obvious chemical shift. In Figure 4A, the peak of O 1s located at 529.54 eV can be indexed to the O^2−^ in the lattice of the samples. The Ni 2p_3/2_ XPS spectra with binding energy of 854.98 eV, corresponding to the Ni 2p_3/2_ peaks of Ni^2+^ and Ni^3+^ located at 854.0 ± 0.2 and 856.0 eV, respectively (Zhang M. et al., 2016). As shown in Figure 4C, the Co 2p_3/2_ binding energy peaks of the samples centers at 780.20 eV, which is agreed with the binding energy of Co^3+^ in LiCoO_2_ (Seteni et al., 2017). The Mn 2p_3/2_ peaks is 641.94 eV, which is consistent with the value of Mn^4+^ (Lou et al., 2017). So the chemical valences of O, Ni, Co, and Mn are−2, +2/+3, +3, and +4, respectively, which indicate that lithium sources cause no effect on valence states.

**Figure 5:**
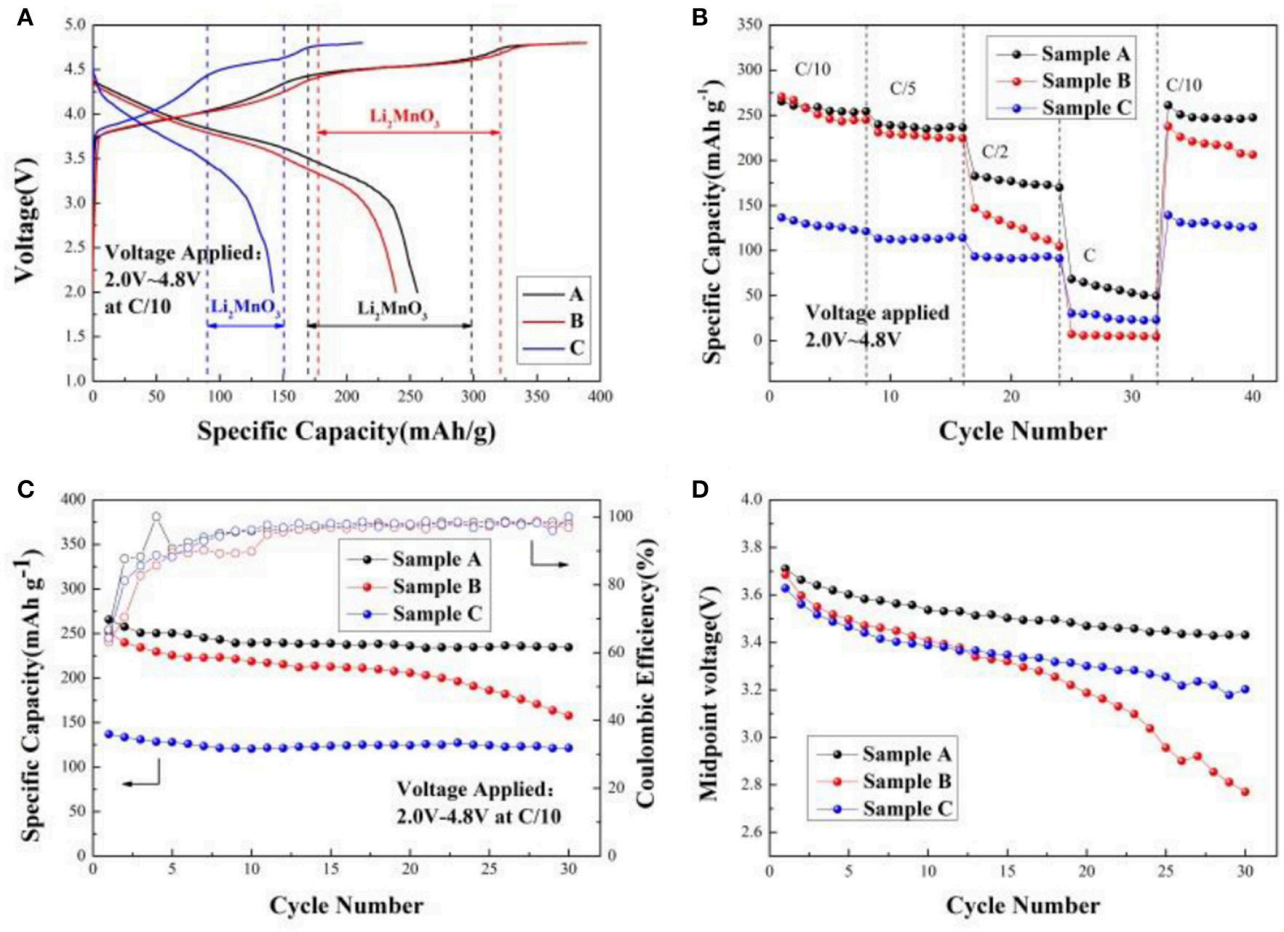
**(A)** Initial charge/discharge curves of the Sample A, B, and C cathodes at C/10 within 2.0–4.8 V; **(B)** Rate performance of Sample A, B, and C cathodes; **(C)** Cycle performance of Sample A, B, and C cathodes at C/10 rate; **(D)** The decay of midpoint voltage of Sample A, B, and C cathodes at C/10 rate.

Figure 5A shows the initial charge/discharge curves of the sample A, B, and C at C/10 within the voltage window of 2.0 and 4.8 V. During initial charge, two plateaus from 3.8 to 4.4 V and from 4.4 to 4.6 V are observed for all samples. The plateau from 3.8 to 4.4 V can be accorded with the oxidation of Ni^2+^/Ni^3+^ to Ni^4+^ and Co^3+^ to Co^4+^, which is in good agreement with the reversible extraction of Li^+^ from LiMO_2_ (M = Ni, Co, Mn) phase (Seteni et al., 2017). Furthermore, the later plateau is attributed to the Li^+^ and O^2−^ irreversible extraction as Li_2_O from the inert Li_2_MnO_3_ phase, resulting in high irreversible capacity (Yang et al., 2016). When the voltage is from 3.8 to 4.4 V during the first charging cycle, the charging mechanism is written as (Lou et al., 2017),

$$\begin{array}{l} 0.5\text{L}{\text{i}}_{2}\text{Mn}{\text{O}}_{3}\cdot 0.5\text{LiN}{\text{i}}_{0.5}\text{C}{\text{o}}_{0.2}\text{M}{\text{n}}_{0.3}{\text{O}}_{2}\overset{charge}{\to }0.5\text{L}{\text{i}}_{2}\text{Mn}{\text{O}}_{3}\cdot 0.5\text{N}{\text{i}}_{0.5}\text{C}{\text{o}}_{0.2}\text{M}{\text{n}}_{0.3}{\text{O}}_{2}+0.5\text{L}{\text{i}}^{+}+0.5{\text{e}}^{-} \end{array}$$

When the voltage is from 4.4 to 4.6 V, the charging mechanism is as follows (Lou et al., 2017),

$$\begin{array}{l} 0.5\text{L}{\text{i}}_{2}\text{Mn}{\text{O}}_{3}\cdot 0.5\text{N}{\text{i}}_{0.5}\text{C}{\text{o}}_{0.2}\text{M}{\text{n}}_{0.3}{\text{O}}_{2}\overset{charge}{\to }0.5\text{Mn}{\text{O}}_{2}\cdot 0.5\text{N}{\text{i}}_{0.5}\text{C}{\text{o}}_{0.2}\text{M}{\text{n}}_{0.3}{\text{O}}_{2}+0.5\text{L}{\text{i}}_{2}\text{O} \end{array}$$

The initial charge/discharge capacity and the coulombic efficiency of the sample A, B, and C are listed in Table 2. Obviously, the discharge capacity of the sample A is much higher than the others. Figure 5B shows rate performance of the Sample A, B, and C at different rates. It can be seen that the discharge capacities of all samples decrease as the rates increased due to the poor conductivity of the material and inert of Li_2_MnO_3_ (Seteni et al., 2017). However, the sample A shows much higher rate property because of smaller primary particle, which can shorten the Li^+^ ions diffusion pathway. The midpoint voltage decay of the samples during cycling at rate of C/10 is shown in Figures 5C,D. The discharge capacities of sample A, B, and C reaches 231.8, 157.7, and 121.0 mAhg^−1^ after 30 cycles at C/10 rate, with capacity retentions of 88.4, 63.8, and 88.6%, respectively. Figure 5D indicates that sample A holds the most stable voltage from 3.71 to 3.43 V during cycling. However, a sudden drop appears for the midpoint voltage of Sample B after 20 cycles. The results indicate sample A keeps the most stable voltage during cycling.

**Table 2:** Initial charge/discharge capacity and the Coulombic efficiency of the sample A, B, and C cathodes.

	**Lithium sources**	**Charge capacity**	**Discharge capacity**	**Initial Coulombic**
		**(mAh g^−1^)**	**(mAh g^−1^)**	**Efficiency (%)**
Sample A	LiOH·H_2_O	382.9	255.6	66.8
Sample B	Li_2_CO_3_	388.9	238.7	61.4
Sample C	CH_3_COOLi·2H_2_O	212.1	142.1	67.0

**Figure 6:**
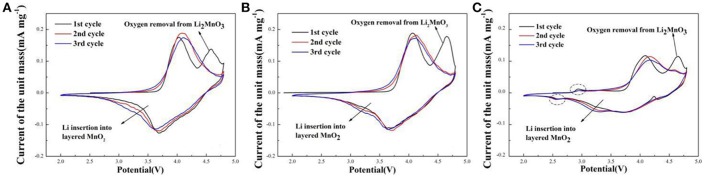
Cyclic voltammetry curves of **(A)** Sample A **(B)** Sample B **(C)** Sample C at the scan rate of 0.1 mV s^−1^.

Cyclic voltammetry curves of sample A, B, and C for the initial three cycles during the voltage range of 2.0–4.8 V at the scan rate of 0.1 mV s^−1^ are shown in Figure 6. The CV curves are similar. There are two oxidation peaks and two reduction peaks in the initial cycle. The oxidation peak at about 4.0 V is the oxidation of Ni^2+^/Ni^3+^ to Ni^4+^ and Co^3+^ to Co^4+^ with the reversible extraction of Li^+^ from LiMO_2_ (M = Ni, Co, Mn) phase (Xiao et al., 2017). The oxidation peak at around 4.6 V corresponds to the Li^+^ and O^2−^ irreversible extraction from the Li_2_MnO_3_ phase. In the following reduction process, the peak at about 3.7 V is the reduction of Ni^4+^ to Ni^2+^/Ni^3+^ and Co^4+^ to Co^3+^, and the peak at around 3.2 V related to Li^+^ insertion into layered MnO_2_ (Seteni et al., 2017). Besides, the peaks of sample A and B (Figures 6A,B) are higher than sample C, indicated that sample A and B have more steady structure. It is also worth mentioning that, there are two peaks at about 2.9 V and 2.5 V for the sample C (Figure 6C), corresponding to oxidation peak and reduction peak, respectively, related to a small amount of spinel structure (Cao et al., 2017).

**Figure 7:**
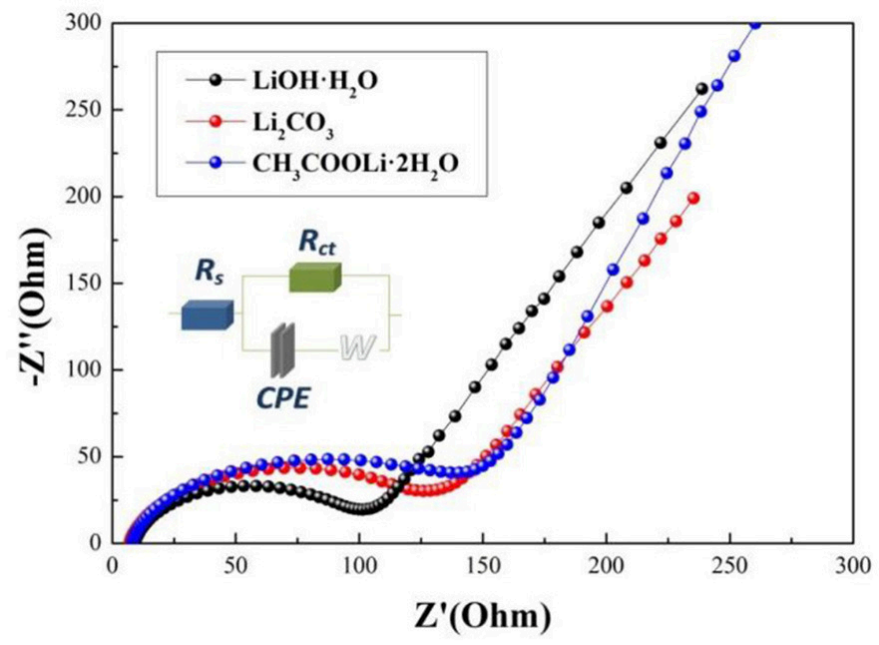
EIS plots of samples using different lithium sources and an equivalent circuit model.

Electrochemical impedance spectroscopy (EIS) can be used to investigate the electrode kinetic process of samples A, B, and C. As shown in Figure 7, The EIS plots consist of a semicircle arc and a straight line. The semicircle arc at high frequency region corresponds to the charge transfer process, and the straight line at low frequency region is the lithium diffusion process. The plots are fitted using the electric equivalent circuit model, as shown in Figure 7. The parameters of the equivalent circuit are listed in Table 3. In the equivalent circuit, R_s_ and R_ct_ represent the solution resistance and charge-transfer resistance, respectively (He et al., 2015). CPE is related to capacitance of the surface layer (Toprakci et al., 2013). Z_W_ represents the Warburg impedance (Xiao et al., 2017) (Z' is the real impedance and Z” is the imaginary impedance). It is observed that the R_s_ and R_ct_ of sample A (5.39 Ω, 87.93 Ω) are smaller than those of sample B (7.968 Ω, 72.08 Ω) and sample C (27.31 Ω, 120 Ω). The results indicate that the solution resistance and charge-transfer resistance of sample A is the smallest, which mainly because sample A prepared by using LiOH·H_2_O owns the smaller primary sizes (Cao et al., 2017). Hence, sample A exhibits the best electrochemical properties.

**Table 3:** EIS fitting values of the samples A, B, and C.

**Samples**	**Sample A**	**Sample B**	**Sample C**
R_s_	5.39	7.968	27.31
R_ct_	72.08	87.93	120

## Conclusions

In summary, 0.5Li_2_MnO_3_·0.5LiNi_0.5_Co_0.2_Mn_0.3_O_2_ materials have been successfully synthesized by using three kinds of lithium sources, LiOH·H_2_O, Li_2_CO_3_, and CH_3_COOLi·2H_2_O, respectively. The effects of morphology, structure, electrochemical performances of the 0.5Li_2_MnO_3_·0.5LiNi_0.5_Co_0.2_Mn_0.3_O_2_ material prepared by using different lithium sources have been investigated. 0.5Li_2_MnO_3_·0.5LiNi_0.5_Co_0.2_Mn_0.3_O_2_ material prepared by using LiOH·H_2_O shows the most uniform morphology with the particle diameters of about 200–300 nm and stable layer structure. It delivers the best electrochemical performances with the initial discharge capacity reaching 255.6 mAh g^−1^ at C/10, and the capacity retention is 88.4% after 30 cycles at C/10. LiOH·H_2_O is the best choice for the synthesis of 0.5Li_2_MnO_3_·0.5LiNi_0.5_Co_0.2_Mn_0.3_O_2_ material compared with Li_2_CO_3_ and CH_3_COOLi·2H_2_O.

## Author contributions

All authors listed have made a substantial, direct and intellectual contribution to the work, and approved it for publication.

### Conflict of interest statement

The authors declare that the research was conducted in the absence of any commercial or financial relationships that could be construed as a potential conflict of interest. The handling Editor declared a shared affiliation, though no other collaboration, with one of the authors, M-ZL.
