# Supplementary material for: Comparative Investigation of 0.5Li2MnO3·0.5LiNi0.5Co0.2Mn0.3O2 Cathode Materials Synthesized by Using Different Lithium Sources
Source: Front Chem. 2018 May 15;6:159. doi: 10.3389/fchem.2018.00159 (PMC5962721; doi:10.3389/fchem.2018.00159)
Supplement: Supplementary file 1 [file Image_1.pdf]

Supporting Information

Comparative investigation of $0.5Li_2MnO_3 \cdot 0.5LiNi_{0.5}Co_{0.2}Mn_{0.3}O_2$ cathode materials synthesized by using different lithium sources

Peng-bo Wang[1], Ming-zeng Luo[1,2], Jun-chao Zheng [1,*], Zhen-jiang He[1], Hui Tong[1] and Wan-jing Yu[1]

1 School of Metallurgy and Environment, Central South University, Changsha, China

2 College of Chemistry and Chemical Engineering, Xiamen University, Xiamen, China

*** Corresponding author:**
Junchao Zheng
E-mail address: jczheng@csu.edu.cn

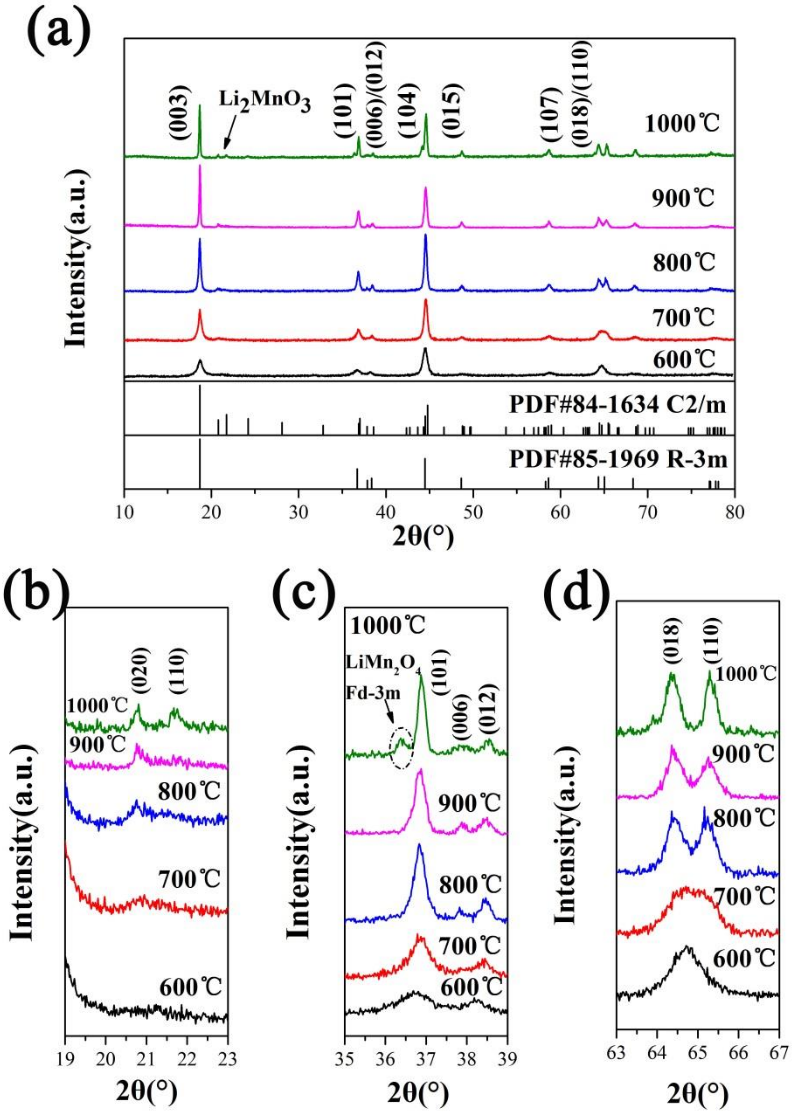

Fig S1. (A) XRD patterns of Sample A using $LiOH{\cdot}H_2O$ sintered at different temperatures and magnified XRD patterns in the 2θ range of **(B)** 19°–23°, **(C)** 35°–39°, **(D)** 63°–67°, respectively.

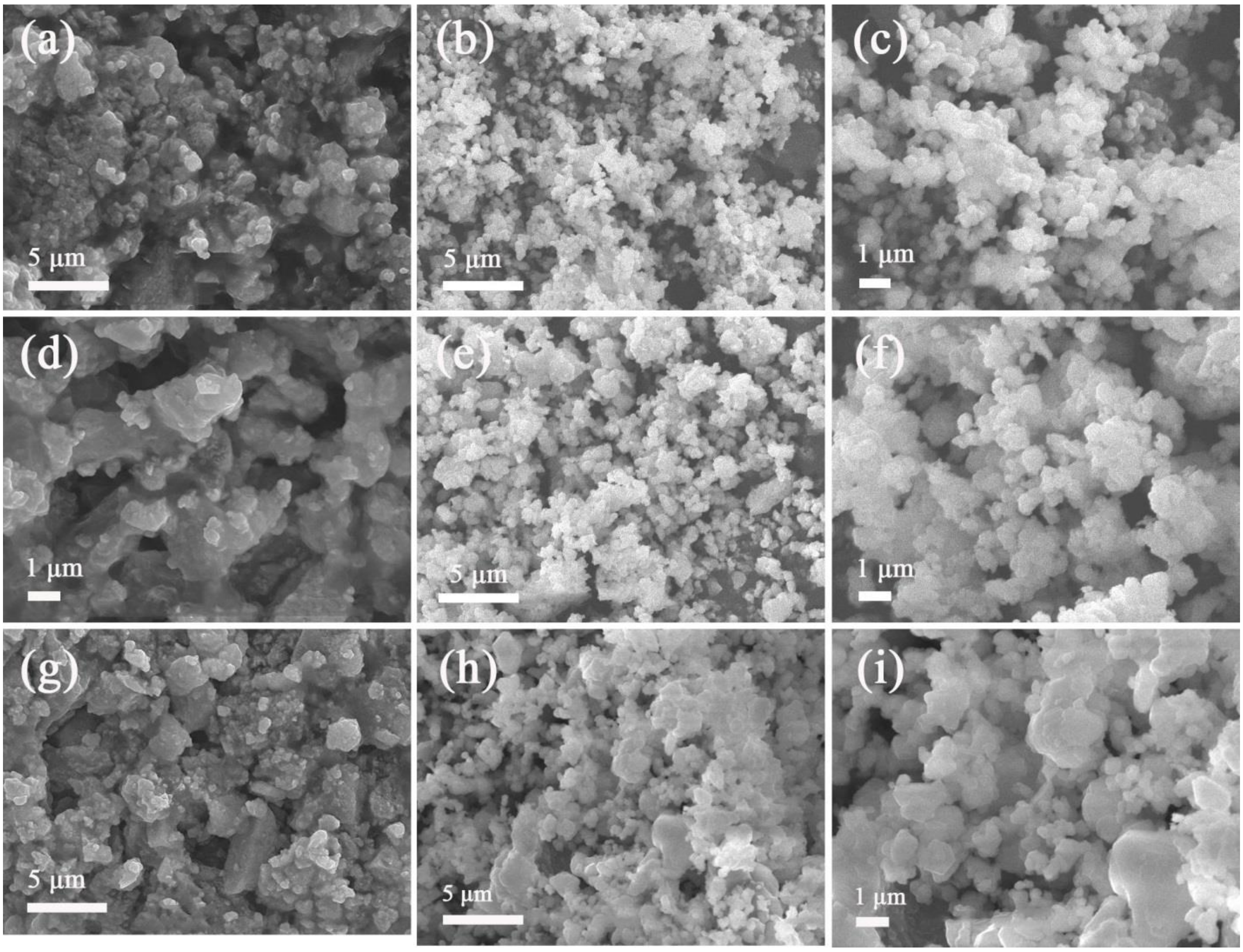

Fig. S2. SEM images of (a) Sample A, (d) Sample B, (f) Sample C before heating processing

and (b-c) Sample A, (e-f) Sample B, (h-i) Sample C
